# Optimal Variational Asymptotic Method for Nonlinear Fractional Partial Differential Equations

**DOI:** 10.1155/2014/847419

**Published:** 2014-10-15

**Authors:** Vipul K. Baranwal, Ram K. Pandey, Om P. Singh

**Affiliations:** ^1^Department of Applied Mathematics, Maharaja Agrasen Institute of Technology, Rohini, Delhi 110086, India; ^2^Department of Mathematics and Statistics, Dr. Hari Singh Gaur University, Sagar 470003, India; ^3^Department of Mathematical Sciences, Indian Institute of Technology, Banaras Hindu University, Varanasi 221005, India

## Abstract

We propose optimal variational asymptotic method to solve time fractional nonlinear partial differential equations. In the proposed method, an arbitrary number of auxiliary parameters *γ*_0_, *γ*_1_, *γ*_2_,… and auxiliary functions *H*_0_(*x*), *H*_1_(*x*), *H*_2_(*x*),… are introduced in the correction functional of the standard variational iteration method. The optimal values of these parameters are obtained by minimizing the square residual error. To test the method, we apply it to solve two important classes of nonlinear partial differential equations: (1) the fractional advection-diffusion equation with nonlinear source term and (2) the fractional Swift-Hohenberg equation. Only few iterations are required to achieve fairly accurate solutions of both the first and second problems.

## 1. Introduction

Nonlinear problems have posed a challenge to the scientific world since long and many scientists and researchers have been working hard to find methods to solve these problems. Quite a remarkable progress has been made to achieve qualitative as well as quantitative solutions of some tough nonlinear problems of significance in the field of physical and biological sciences, as well as in engineering and technology. There are several analytical methods, such as homotopy analysis method (HAM) [1], homotopy perturbation method (HPM) [2], Adomian decomposition method (ADM) [3], variational iteration method (VIM) [4], and a new iterative method [5], are available to solve nonlinear fractional partial differential equations.

The aim of the present paper is to propose an optimal variational asymptotic method (OVAM) to solve the following initial value nonlinear time fractional PDE:

(1) Ly+Ny=g(x,t), x∈Ω=[a,b]⊂R,  t>0,

with the initial conditions

(2) y(x,0)=f0(x),y′(x,0)=f1(x),…,y(n−1)(x,0)=fn−1(x),

where *L* = ∂^*α*^/∂*t* ^*α*^, *n* − 1 < *α* ≤ *n*, and *N* is the nonlinear part of the fractional PDE, and *g*(*x*, *t*) is the source term. The fractional derivatives are taken in the Caputo sense. The proposed method is the generalization of the method given in [5].

To test the method, we apply it to solve two important classes of nonlinear partial differential equations: (1) the fractional advection-diffusion equation with nonlinear source term and (2) the fractional Swift-Hohenberg equation.

## 2. Analysis of the Method

The variational iteration method (VIM) is a well-established iteration method [6]. The main drawbacks of the solution obtained by standard VIM are that it is convergent in a small region and handling the nonlinear terms is a difficult task. To enlarge the convergence region and remove the difficulty of handling the nonlinear terms, we propose a new iterative method based on a new modification of VIM, which is different from the other previous modifications.

First we generalize the correction functional of our earlier work [5] by a new generalized correction functional (3). This is achieved by introducing in it auxiliary parameters *γ*_0_, *γ*_1_, *γ*_2_,… (as these parameters are used to control the region of convergence of the solution series, they are also called convergence control parameters) and auxiliary functions *H*_0_(*x*), *H*_1_(*x*), *H*_2_(*x*),…. Taking *γ*_0_ = *γ*, *H*_0_(*x*) = *H*(*x*), and *γ*_1_ = *γ*_2_ = *γ*_3_ = ⋯ = 0, we get the correction functional of [5]. Further, we express the nonlinear term *Ny*_*n*_ in terms of the Adomian polynomials.

To illustrate the method, we consider the general form of the initial value fractional partial differential equation described by (1)-(2).

The new generalized correction functional for (1) is constructed as follows:

(3) yn+1(x,t)=yn(x,t)+Jtα ×[∑j=0n(γj  Hj(x)λ{∑j=0n(γj  Hj(x)Lyn−Ly~n+∑j=0n(γj  Hj(x) iiiiiiiiiiiiiiiiiiiiiiiiiiiiiiiiiiiii×(Ly~n−j(x,t)mmmmmmmmmmmmmm+Ny~n−j(x,t) iiiiiiiiiiiiiiiiiiiiiiiiiiiiiiiiiiiiiii−G~n−j(x,t)))∑j=0n(γj  Hj(x)}],

(4) yn+1(x,t)=yn(x,t)+1Γ(α) ×∫0t(t−s)α−1λ[∑j=0n{γj  Hj(x)Lyn−Ly~niiiiiiiiiiiiiiiiiiiiiiiiiiiiiii+∑j=0n{γj  Hj(x)mmmmmmmmmmmmmm×(Ly~n−j(x,s)mmmmmmmmmmmmmmm+Ny~n−j(x,s)iiiiiiiiiiiiiiiiiiimmmmmmmmm−G~n−j(x,s))}∑j=0n{γj  Hj(x)]ds,

where *J*_*t*_^*α*^ is the Riemann-Liouville fractional integral operator of order *α* with respect to the variable *t* and *λ* is general Lagrange multiplier which is identified optimally via variational theory, the subscript *n* denotes the *n*th approximation, and ${\tilde{y}}_{n}$ is considered as restricted variation; that is, $\delta {\tilde{y}}_{n}=0$. The sequence *G*_*n*_(*x*, *t*) is defined as follows.

Writing *g*(*x*, *t*) = ∑_*i*=0_^*m*^ *g*_*i*_(*x*, *t*), a sequence *G*_*n*_(*x*, *t*) [5] is constructed with suitably chosen support, as

(5) $$\begin{array}{c} G_{n}\left(x,t\right)=\overset{m}{\underset{i=0}{\sum }}\chi_{n-i+2}\;g_{i}\left(x,t\right),\;\text{where}\;\chi_{n}=\{\begin{array}{cc} 0, & n\leq 1,\\ 1, & n>1. \end{array} \end{array}$$

To determine the optimal value of Lagrange multiplier *λ* via variational theory we use the following proposition.

**Proposition 1 .**

Consider *δ*[(1/Γ(*n*))∫_0_^*t*^(*t*−*τ*)^*n*−1^(∂^*n*^ *u*_*k*_(*x*, *τ*)/∂*τ* ^*n*^)*dτ*] = *δu*_*k*_(*x*, *t*) [5].

Taking *α* = *n* in (4) and using Proposition 1, we obtain

(6) δyn+1(x,t)=δyn(x,t) +δ(1Γ(α)∫0t(t−s)α−1λ  iiiiiiiiiiiiiiii×{∑j=0n{γj  Hj(x)Lyn−Ly~nkkkkkkkkkkkkkkkki+∑j=0n{γjHj(x)kkkkkkkkkkkkkkkkkkkkkk×(Ly~n−j(x,s)kkkkkkkkkkkkkkkkkkkkkk  +Ny~n−j(x,s)iiiiiiiiiiiiiiiiiiiiiiiiiiiiiiiiiiiiiiiiiiiiiiiiii−G~n−j(x,s))}1Γ(α)ef}ds)=δyn(x,t)  +δ(1Γ(n)∫0tλ(t−s)n−1  dnyndsn  ds)=(1+λ(s)|s=t)δyn(x,t)  −∫0tλ′(s)δyn(x,s)ds,

giving 1 + *λ*(*s*)|_*s*=*t*_ = 0 and *λ*′(*s*)|_*s*=*t*_ = 0. Thus, we have *λ*(*t*) = −1.

Substituting *λ* = −1 and discarding the added and subtracted terms *Ly*_*n*_ in (3) we get

(7) yn+1(x,t)=yn(x,t) −Jtα[∑j=0n{γjHj(x)(Lyn−j(x,t) iiiiiiiiiiiiiiiiiiiiiiiiiiiii +Nyn−j(x,t) iiiiiiiiiiiiiiiiiiiiiiiiii−Gn−j(x,t))}∑j=0n{γj  Hj(x)(Lyn−j(x,t)],mmmmmmmmmmmmn=0,1,2,….

In our proposed algorithm, computing (7) for a given problem will be referred to as the first step.

Writing

(8) $$\begin{array}{c} y_{n}\left(x,t\right)=\overset{n}{\underset{m=0}{\sum }}u_{m}\left(x,t\right), \end{array}$$

where

(9) u0(x,t)=y(x,0)+ty′(x,0)+t2y′′(x,0) +⋯+tn−1y(n−1)(x,0)+Jtα(g(x,t))=∑j=0n−1fj(x)+Jtα(g(x,t)),

we get the series representation of the solution *y*(*x*, *t*) as

(10) $$\begin{array}{c} y\left(x,t\right)=\underset{n\to \infty }{\lim}y_{n}\left(x,t\right)=\underset{n\to \infty }{\lim}\overset{n}{\underset{m=0}{\sum }}u_{m}\left(x,t\right). \end{array}$$

The nonlinear term *Ny*_*n*_(*x*, *t*) is expanded in terms of Adomian's polynomials as

(11) $$\begin{array}{c} Ny_{n}\left(x,t\right)=N\left(\overset{n}{\underset{m=0}{\sum }}u_{m}\left(x,t\right)\right)=\overset{n}{\underset{m=0}{\sum }}A_{m}\left(u_{0},u_{1},\ldots ,u_{m}\right), \end{array}$$

where *A*_*m*_'s are Adomian's polynomials which are calculated by the algorithm (12) constructed by Adomian [7]:

(12) $$\begin{array}{c} A_{n}\left(u_{0},u_{1},\ldots ,u_{n}\right)=\frac{1}{n!}{\left[\frac{d^{n}}{d\lambda^{n}}N\left(\overset{n}{\underset{k=0}{\sum }}\lambda^{k}u_{k}\right)\right]}_{\lambda =0},\;n\geq 0. \end{array}$$

Evaluation of the nonlinear term *Ny*_*n*_(*x*, *t*) by (12) will be referred to as the second step.

Combining the first and second step, the new generalized correction functional (7) becomes

(13) un+1(x,t)=−Jtα[∑j=0n{γjHj(x)(−Gn−j(x,t)L(∑m=0n−jum(x,t))iiiiiiiiiiiiiiiiiiiiiiiiiiiiiiiiiiiiiiiii+∑m=0n−jAm(u0,u1,…,um)lllllllllllllllllllllllllllllllllllliiiiiiii−Gn−j(x,t)(∑m=0n−jum(x,t)))γj  Hj(x)(L}∑j=0n{γj  Hj(x)(L],iiiiiiiiiiiiiiiiiiiiiiiiiiiiiiiiiiiiiiiiiiiiiiin=0,1,2,….

Equation (13) can also be written as

(14) u1(x,t)=−γ0H0(x)Jtα ×[Lu0(x,t)+A0(u0)−G0(x,t)],un+1(x,t)=un(x,t)−Jtα ×[∑j=0n{γjHj(x)iiiiiiiiiiiiiiiii×(Lun−j(x,t)+An−jiiiiiiiiiiiiiiiiiiiiiii×(u0,u1,…,un−j)iiiiiiiiiiiiiii iiiiii−(Gn−j(x,t)iiiiiiiiiiiiiiiiiiiii iimm−χn−j+1Gn−j−1(x,t)))}∑j=0n{γj  Hj(x)],iiiiiiiiiiiiiiiiiiiiiiiiiiiiiiiiiiiiiiiiiiiiiiiin=1,2,….

Combining the above two equations, we get

(15) un+1(x,t)=χn+1un(x,t)−Jtα ×[∑j=0n{−χn−j+1Gn−j−1(x,t)))γjHj(x)iiiiiiiiiiiiiii×(Lun−j(x,t)iiiiiiiiiiiiiiiiiii+An−j(u0,u1,…,un−j)iiiiiiiiiiiiiiiiiii−(Gn−j(x,t)iiiiiiiiiiiiiiiiiiiiiiiii−χn−j+1Gn−j−1(x,t)))}∑j=0n{γj  Hj(x)].

From (15), we calculate the various *u*_*n*_(*x*, *t*) for *n* ≥ 1 and substituting these values in (10), we obtain the analytical solution of (1).

Truncating the solution series (10) at level *m* = *n*, the approximate solution at level *n* is given by

(16) y~n(x,t,γ0,γ1,…,γn−1)=u0(x,t) +∑j=1nui(x,t,γ0,γ1,…,γj−1).

The values of *γ*_*j*_'s are still to be found.

Substituting (16) into (1), one gets the following residual:

(17) Rn(x,t,γ0,γ1,…,γn−1) =L(y~n(x,t,γ0,γ1,…,γn−1))  +N(y~n(x,t,γ0,γ1,…,γn−1))−g(x,t).

If *R*_*n*_ = 0, then ${\tilde{y}}_{n}$ will be the exact solution. Generally such a case will not arise for nonlinear and fractional problems. There are several methods like Galerkin's method, Ritz method, least squares method, and collocation method to find the optimal values of *γ*_0_, *γ*_1_, *γ*_2_,…. We apply the method of least squares to compute the optimal values of these auxiliary parameters.

At the* n*th-order of approximation, we define the exact square residual error *J*_*n*_(*γ*_0_, *γ*_1_,…, *γ*_*n*−1_) as

(18) $$\begin{array}{c} J_{n}\left(\gamma_{0},\gamma_{1},\ldots ,\gamma_{n-1}\right)=\overset{t_{1}}{\underset{t_{0}}{\int }}\overset{}{\underset{\Omega }{\int }}R_{n}^{2}\left(x,t,\gamma_{0},\gamma_{1},\ldots ,\gamma_{n-1}\right)dx\;dt. \end{array}$$

Thus, at the given level of approximation *n*, the corresponding optimal values of convergence control parameters *γ*_0_, *γ*_1_,…, *γ*_*n*−1_ are obtained by minimizing the *J*_*n*_ which corresponds to the following set of *n* algebraic equations:

(19) $$\begin{array}{c} \frac{\partial J_{n}}{\partial \gamma_{0}}=0,\frac{\partial J_{n}}{\partial \gamma_{1}}=0,\ldots ,\frac{\partial J_{n}}{\partial \gamma_{n-1}}=0. \end{array}$$

The optimal values of *γ*_0_, *γ*_1_,…, *γ*_*n*−1_ so obtained, when substituted in equation (16) gives the approximate solution at level *n*.

The novelty of our proposed algorithm is that (1) a new generalized correction functional (15) is constructed by introducing auxiliary parameters *γ*_0_, *γ*_1_, *γ*_2_,…, auxiliary functions *H*_0_(*x*), *H*_1_(*x*), *H*_2_(*x*),…, and expanding the nonlinear term as series of Adomian polynomial in the correction functional of the standard VIM and (2) the values of auxiliary parameters *γ*_0_, *γ*_1_, *γ*_2_,…, *γ*_*n*−1_ are obtained optimally by using (16)–(19).

## 3. Applications

Now we apply our proposed method to solve the following two problems in Sections 3.1 and 3.2.

### 3.1. Fractional Advection-Diffusion Equation (FADE) with Nonlinear Source Term

Advection-diffusion equation (ADE) describes the solute transport due to combined effect of diffusion and convection in a medium. It is a partial differential equation of parabolic type, derived on the principle of conservation of mass using Fick's law. Due to the growing surface and subsurface hydro environment degradation and the air pollution, the advection-diffusion equation has drawn significant attention of hydrologists, civil engineers, and mathematical modellers. Its analytical/numerical solutions along with an initial condition and two boundary conditions help to understand the contaminant or pollutant concentration distribution behaviour through an open medium like air, rivers, lakes, and porous medium like aquifer, on the basis of which remedial processes to reduce or eliminate the damages may be enforced. It has wide applications in other disciplines too, like soil physics, petroleum engineering, chemical engineering, and biosciences. In 2002, Inc and Cherruault [9] applied Adomian decomposition method to solve nonlinear convection- (advection-) diffusion equation.

The fractional order forms of the ADE are similarly useful. The most important advantage of using fractional order differential equation in mathematical modelling is their nonlocal property. It is a well-known fact that the integer order differential operator is a local operator whereas the fractional order differential operator is nonlocal in the sense that the next state of the system depends not only upon its current state but also upon all of its proceeding states. In the last decade, many authors have made notable contribution to both theory and application of fractional differential equations in areas as diverse as finance [10], physics [11, 12], control theory [13], and hydrology [14, 15]. Several papers have been written [16, 17] to show the equivalence between the transport equations using fractional order derivatives and some heavy-tailed motions, thus extending the predictive capability of models built on the stochastic process of Brownian motion, which is basis for the classical ADE. The motion can be heavy-tailed, implying extremely long-term correlation and fractional derivatives in time and/or space.

In recent past several papers [8, 14, 15, 18, 19] have been written to solve FADE. In 2007, Momani [8] proposed an algorithm to solve the following FADE with nonlinear source term:

(20) ∂αy∂tα=∂2y∂x2−c∂y∂x+Ψ(y)+f(x,t), iimm0<x<1,  t≥0,  0<α≤1,

(21) $$\begin{array}{c} y\left(0,t\right)=h_{1}\left(t\right),\;\;t\geq 0, \end{array}$$

(22) $$\begin{array}{c} \frac{\partial y\left(1,t\right)}{\partial x}=h_{2}\left(t\right),\;t\geq 0, \end{array}$$

(23) $$\begin{array}{c} y\left(x,0\right)=g\left(x\right),\;0\leq x\leq 1, \end{array}$$

where Ψ(*y*) is some reasonable nonlinear function of *y* which is chosen as a potential energy, *c* is a constant, and *α* is a parameter describing the order of the time-fractional derivative. The fractional derivative is considered in the Caputo sense. In [8], the author solved the above problem by taking Ψ  (*y*) = *y*(∂^2^ *y*/∂*x* ^2^) − *y* ^2^ + *y*, *c* = 1, *f*(*x*, *t*) = 0, *h*_1_(*t*) = *e* ^*t*^, *h*_2_(*t*) = *e* ^*t*+1^, and *g*(*x*) = *e* ^*x*^. With these values (20)–(23) are reduced to

(24) ∂αy∂tα=∂2y∂x2−∂y∂x+y∂2y∂x2−y2+y,  0<x<1,  t≥0,  0<α≤1,

(25) $$\begin{array}{c} y\left(0,t\right)=e^{t},\;\;t\geq 0, \end{array}$$

(26) $$\begin{array}{c} \frac{\partial y\left(1,t\right)}{\partial x}=e^{t+1},\;t\geq 0, \end{array}$$

(27) $$\begin{array}{c} y\left(x,0\right)=e^{x},\;0\leq x\leq 1, \end{array}$$

with exact solution*y*(*x*, *t*) = *e* ^*x*+*t*^ for *α* = 1.

As the first illustration of our proposed method, we apply it to solve the FADE described by (24)–(27).

Taking *H*_*i*_(*x*) = −1, *i* = 0,1, 2,…, *n*, *t*_0_ = 0, *t*_1_ = 1, *g*_*j*_(*x*, *t*) = 0, *j* = 0,1, 2,…, *m*, and *u*_0_(*x*, *t*) = *e* ^*x*^ and applying the proposed algorithm, we obtain the correction functional (15) for (24) as

(28) un+1(x,t)=χn+1un(x,t)+  Jtα ×[∑i=0n{γi(∂α∂tαun−i(x,t)iiiiiiiiiiiiiiiiiiii −∂2∂x2un−i(x,t)+∂∂xun−i(x,t)iiiiiiiiiiiiiiiiiiii −An−i(u0,u1,…,un−i)iiiiiiiiiiiiiiiiiiii +Bn−i(u0,u1,…,un−i)iiiiiiiiiiiiiiiiiiii −un−i(x,t)∂α∂tαun−i(x,t))}∑i=0n{γi(∂α∂tαun−i(x,t)],

where

(29) An(u0,u1,…,un) =1n![dndλn{(∑k=0nλkuk)·∂2∂x2(∑k=0nλkuk)}]λ=0,iiiiiiiiiiiiiiiiiiiiiiiiiiiiiiiiiiiiiiiiiiiiiiiiiiiiiiiiiiiiiiiiin≥0,Bn(u0,u1,…,un)=1n![dndλn(∑k=0nλkuk)2]λ=0,iiiiiiiiiiiiiiiiiiiiiiiiiiiiiiiiiiiiiiiiiiiiiiiiiiiiiiiiiiiin≥0.

Solving (28) and using (29), we get the various *u*_*n*_(*x*, *t*) as

(30) $$\begin{array}{c} u_{1}\left(x,t\right)=-\gamma_{0}e^{x}\frac{t^{\alpha }}{\Gamma \left(1+\alpha \right)}, \end{array}$$

(31) u2(x,t)=−γ0(1+γ0)extαΓ(1+α) −γ1extαΓ(1+α)+γ02  ext2αΓ(1+2  α),….

Substituting the above iterations in (16) and taking *n* = 5, the 5th order approximate solution for (24)–(27) is obtained as

(32) $$\begin{array}{c} {\tilde{y}}_{5}\left(x,t,\gamma_{0},\gamma_{1},\ldots ,\gamma_{4}\right)=u_{0}\left(x,t\right)+\overset{5}{\underset{i=1}{\sum }}u_{i}\left(x,t,\gamma_{0},\gamma_{1},\ldots ,\gamma_{i}\right). \end{array}$$

From (17) the 5th order residual is

(33) R5(x,t,γ0,γ1,…,γ4)=∂α∂tαy~5−∂2∂x2y~5 +∂∂xy~5−y~5∂2∂x2y~5+y~52−y~5.

To determine the optimal values of *γ*_0_, *γ*_1_,…, *γ*_4_, we minimize the square residual error given in (18). As discussed in [20], computing *J*_5_(*γ*_0_, *γ*_1_,…, *γ*_4_) directly with symbolic computational software is impractical. Thus, we approximate (18) using Gaussian Legendre quadrature with twenty nodes. The optimal values of *γ*_0_, *γ*_1_,…, *γ*_4_ for all the values of *α* considered are obtained by minimizing (18) using the Mathematica function Minimize and are given in Table 1. Before approximating (18) using Gaussian Legendre quadrature with twenty nodes, we replace *x* and *t* by (1 + *x*)/2 and (1 + *t*)/2, respectively.

We define the absolute errors $E_{n}\left(\gamma_{0},\gamma_{1},\gamma_{2},\ldots ,\gamma_{n-1}\right)=\left|y_{\text{exact}}\left(x,t\right)-{\tilde{y}}_{n}\left(x,t,\gamma_{0},\gamma_{1},\gamma_{2},\ldots ,\gamma_{n-1}\right)\right|$ and *E*_*n*_^*o*^ = *E*_*n*_(*γ*_0_, *γ*_1_, *γ*_2_,…, *γ*_*n*−1_), evaluated at the optimal values of the convergence control parameters *γ*_*j*_'s.

Table 1 lists that the optimal values of *γ*_0_, *γ*_1_,…, *γ*_4_ and *J*_5_.  Table 2 shows the comparison between the fifth order solution obtained by our method for the optimal values of *γ*_0_, *γ*_1_,…, *γ*_4_ given in Table 1 and the fifteenth order solution given in [8] for different values of *α*. From Table 2 we see that as the value of *α* moves from 1 to 0 the solution of our method differs more from that of [8], whereas for *α* = 1 these are in complete agreement.

**Figure 1:**
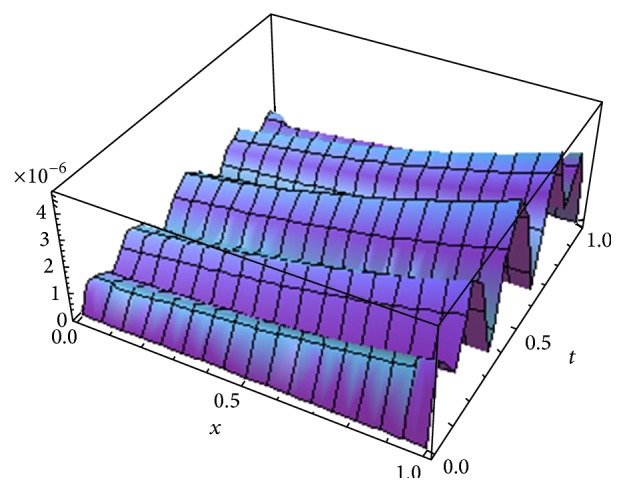
The error *E*_5_^*o*^ for *α* = 1.

**Figure 2 fig2:**
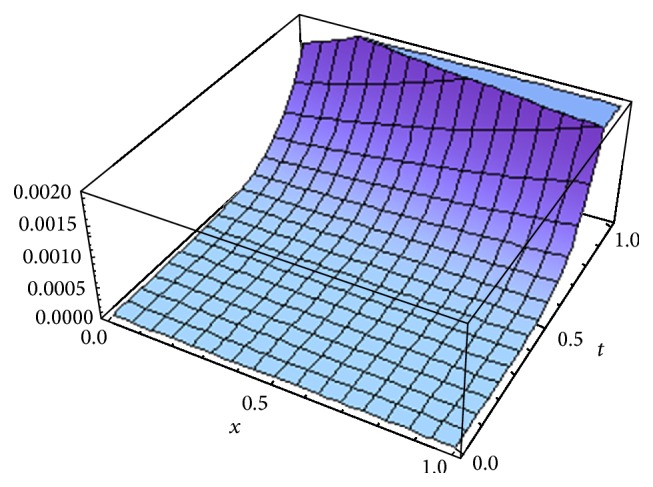
The fifth order error, Momani [8] for *α* = 1.

**Figure 3:**
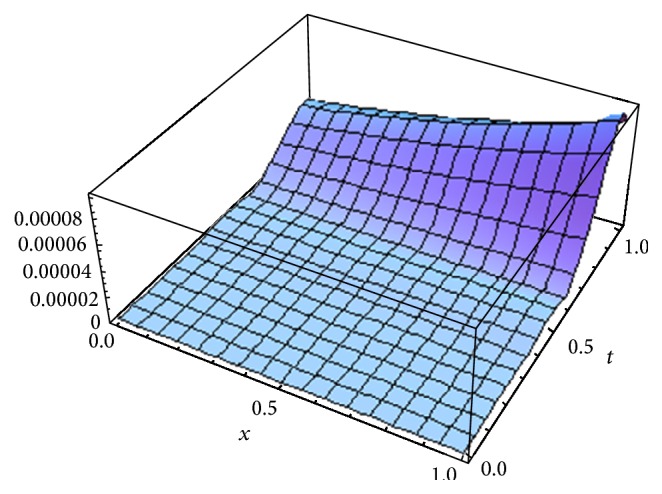
The error *E*_5_ [5] for *α* = 1.

**Figure 4:**
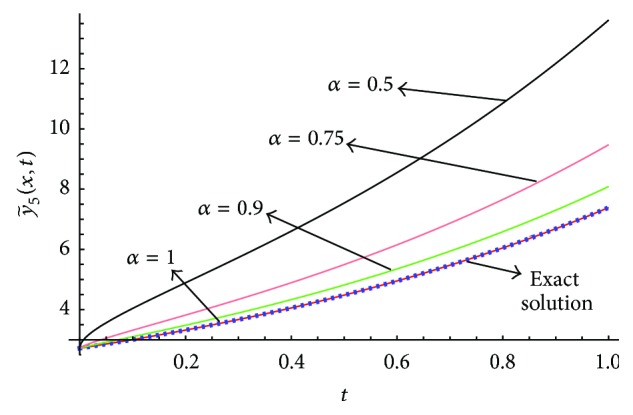
Cross section of ${\tilde{y}}_{5}\left(x,t\right)$ at *x* = 1 for different *α*.

Figure 1 shows the error *E*_5_^*o*^ for *α* = 1, whereas Figure 2 shows the error obtained in [8] using fifth term approximate solution given by HPM for *α* = 1.  Figure 3 shows the error obtained by fifth order approximate solution using the new iterative method [5] by taking *u*_0_(*x*, *t*) = *e* ^*x*^ and *H*(*x*) = −1 for *α* = 1. From Figures 1 [Fig fig2]–3 we conclude that the fifth order approximate solution obtained by our method is more accurate as compared to the same order solutions obtained by the new iterative method [5] and HPM [8].  Figure 4 shows the cross section of approximate solution ${\tilde{y}}_{5}(x,t)$ at *x* = 1 for different values of *α* and the corresponding optimal values of *γ*_0_, *γ*_1_,…, *γ*_4_ given in Table 1.

### 3.2. Fractional Swift-Hohenberg Equation

Density gradient-driven fluid convection arises in geophysical fluid flows in the atmosphere, oceans, and in the earth's mantle. The Rayleigh-Benard convection is a prototype model for fluid convection, aiming at predicting spatiotemporal convection patterns. The mathematical model for the Rayleigh-Benard convection involves the Navier-Stokes equations coupled with the transport equation for temperature. When the Rayleigh number is near the onset of convection, the Rayleigh-Benard convection model may be approximately reduced to an amplitude or order parameter equation, as derived by Swift and Hohenberg [21].

The Swift-Hohenberg (SH) equation is defined as

(34) $$\begin{array}{c} \frac{\partial y}{\partial t}=\mu y-{\left(1+\frac{\partial^{2}}{\partial x^{2}}\right)}^{2}y-y^{3}, \end{array}$$

where *μ* ∈ *ℝ* is a parameter. It is a simple model for the Rayleigh-Benard convective instability of roll waves [22]. The Swift-Hohenberg (SH) equation has numerous important applications in the different branches of Physics such as Taylor-Couette flow [21, 23] and in the study of lasers [24]. It also plays a vital role in the study of pattern formation [25] and as a model equation for a large class of higher-order parabolic equations arises in a wide range of applications, for example, as the extended Fisher-Kolmogorov equation in statistical mechanics [26], as well as a sixth order equation introduced by Caginalp and Fife [27] in phase field models [28]. In 1995, Caceres [29] considered the Swift-Hohenberg equation for piece-wise constant potentials and found the eigenvalues for it. Later in 2002, Christrov and Pontes [30] gave the numerical scheme for Swift-Hohenberg equation with strict implementation of Lyapunov functional. Peletier and Rottschäfer [31] in 2003 studied the large time behaviour of solution of Swift-Hohenberg equation. Two years later Day et al. [32] in 2005 also provided the numerical solution to the Swift-Hohenberg equation. Some other research papers related to SH equation were published by different authors [33, 34]. Akyildiz et al. [35] in 2010 have solved the Swift-Hohenberg equation by homotopy analysis method for the standard motion. Recently, in 2011, Khan et al. [36] gave the approximate solution of SH equation with Cauchy-Dirichlet condition. In the same year Khan et al. [37] have solved the Swift-Hohenberg equation with fractional time derivative using homotopy perturbation method and differential transform method, whereas Vishal et al. [38] have solved the fractional Swift-Hohenberg equation using homotopy analysis method.

We consider the following time fractional Swift-Hohenberg equation [37, 38]:

(35) ∂αy∂tα+2∂2y∂x2+∂4y∂x4+(1−μ)y+y3=0,iiiiiiiiiiiiiiiiii0<x<l,    t>0,    0<α≤1,

with the initial condition

(36) $$\begin{array}{c} y\left(x,0\right)=\frac{1}{10}\sin\left(\frac{\pi x}{l}\right), \end{array}$$

and boundary conditions

(37) $$\begin{array}{c} y\left(x,t\right)=0,\;\frac{\partial^{2}y\left(x,t\right)}{\partial x^{2}}=0\;\;\text{at}\;\;x=0,l,\;\;t>0. \end{array}$$

Taking *H*_*i*_(*x*) = −1, *i* = 0,1, 2,…, *n*, *t*_0_ = 0, *t*_1_ = 1, *g*_*j*_(*x*, *t*) = 0, *j* = 0,1, 2,…, *m*, and *u*_0_(*x*, *t*) = (1/10)sin(*πx*/*l*) and applying the proposed algorithm, we obtain the correction functional (15) for (21) as

(38) un+1(x,t)=χn+1un(x,t)+Jtα ×[∑i=0n{+An−i(u0,u1,…,un−i)∂α∂tαun−i)γi(∂α∂tαun−i(x,t) iiiiiiiiiiiiiiiiiii+2∂2∂x2un−i(x,t) iiiiiiiiiiiiiiiiiii+∂4∂x4un−i(x,t) iiiiiiiiiiiiiiiiiii+(1−μ)un−i(x,t) iiiiiiiiiiiiiiiiiii+An−i(u0,u1,…,un−i)∂α∂tαun−i)}ef22∑i=0n{γi],

where

(39) An(u0,u1,…,un)=1n![dndλn{(∑k=0nλkuk)3}]λ=0,iiiiiiiiiiiiiiiiiiiiiiiiiiiiiiiiiiiiiin≥0.

Solving (38) and using (39), we get the various *u*_*n*_(*x*, *t*) as

(40) u1(x,t)=γ0(−15l2π2sin(πxl)+110l4π4sin(πxl)  m+110(1−μ)sin(πxl)+11000sin(πxl)3) ×tαΓ(1+α),….

Substituting the above iterations in (16) and taking *n* = 3, the 3rd order approximate solution for (35)–(37) is obtained as

(41) $$\begin{array}{c} {\tilde{y}}_{3}\left(x,t,\gamma_{0},\gamma_{1},\gamma_{2}\right)=u_{0}\left(x,t\right)+\overset{3}{\underset{i=1}{\sum }}u_{i}\left(x,t,\gamma_{0},\gamma_{1},\ldots ,\gamma_{i}\right). \end{array}$$

From (17), the 3rd order residual is

(42) R3(x,t,γ0,γ1,γ2)=∂α∂tαy~3+2∂2∂x2y~3 +∂4∂x4y~3+(1−μ)y~3+y~33.

As discussed in Section 3.1, we first replace *x* and *t* by *l*(1 + *x*)/2 and (1 + *t*)/2, respectively, and then approximate (18) using Gaussian Legendre quadrature with twenty nodes. The optimal values of *γ*_0_, *γ*_1_, *γ*_2_ for all the values of *α*, *l*, and *μ* considered are obtained by minimizing (18) using the Mathematica function Minimize and are given in Table 3. From Table 3 we see that the square residual error *J*_3_ for *l* = 10, *μ* = 0.6, and *α* = 1, 0.75, 0.5  , respectively, obtained by our algorithm is smaller than that obtained by Vishal et al. [38] using eighth order approximate solution of HAM as given in Tables 1 [Table tab2]–3 [38].

**Figure 5:**
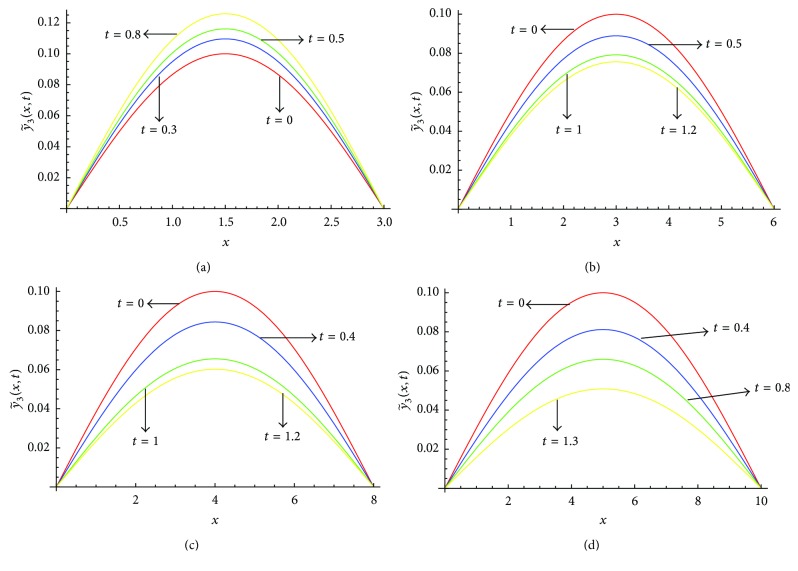
(a) Profiles of ${\tilde{y}}_{3}(x,t)$ versus *x* at *α* = 1, *μ* = 0.3 for *l* = 3 on the corresponding values of *γ*_0_, *γ*_1_, *γ*_2_ as given in Table 3. (b) Profiles of ${\tilde{y}}_{3}(x,t)$ versus *x* at *α* = 1, *μ* = 0.3 for *l* = 6 on the corresponding values of *γ*_0_, *γ*_1_, *γ*_2_ as given in Table 3. (c) Profiles of ${\tilde{y}}_{3}(x,t)$ versus *x* at *α* = 1, *μ* = 0.3 for *l* = 8 on the corresponding values of *γ*_0_, *γ*_1_, *γ*_2_ as given in Table 3. (d) Profiles of ${\tilde{y}}_{3}(x,t)$ versus *x* at *α* = 1, *μ* = 0.3 for *l* = 10 on the corresponding values of *γ*_0_, *γ*_1_, *γ*_2_ as given in Table 3.

**Figure 6:**
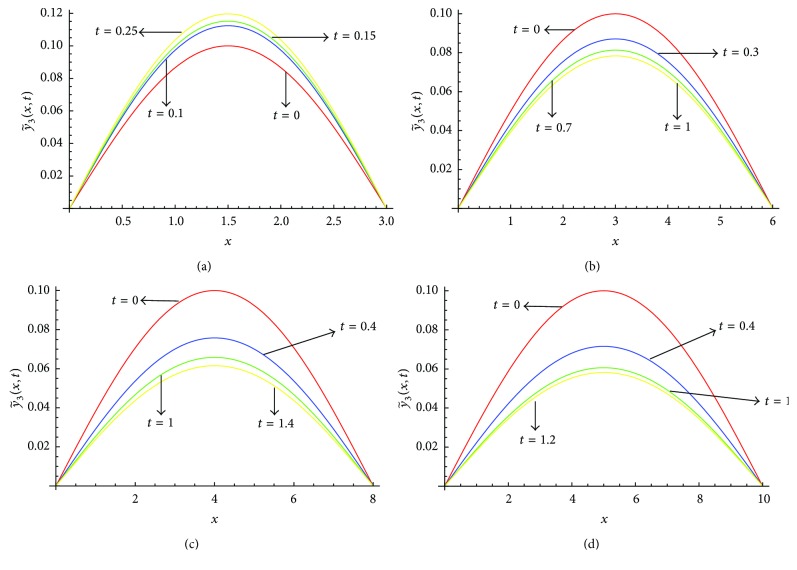
(a) Profiles of ${\tilde{y}}_{3}(x,t)$ versus *x* at *α* = 0.5, *μ* = 0.3 for *l* = 3 on the corresponding values of *γ*_0_, *γ*_1_, *γ*_2_ as given in Table 3. (b) Profiles of ${\tilde{y}}_{3}(x,t)$ versus *x* at *α* = 0.5, *μ* = 0.3 for *l* = 6 on the corresponding values of *γ*_0_, *γ*_1_, *γ*_2_ as given in Table 3. (c) Profiles of ${\tilde{y}}_{3}(x,t)$ versus *x* at *α* = 0.5, *μ* = 0.3 for *l* = 8 on the corresponding values of *γ*_0_, *γ*_1_, *γ*_2_ as given in Table 3. (d) Profiles of ${\tilde{y}}_{3}(x,t)$ versus *x* at *α* = 0.5, *μ* = 0.3 for *l* = 10 on the corresponding values of *γ*_0_, *γ*_1_, *γ*_2_ as given in Table 3.

Figures 5(a)–5(d) show the solution profile ${\tilde{y}}_{3}(x,t)$ versus *x* for *l* = 3, *l* = 6, *l* = 8, and *l* = 10, respectively, at *α* = 1, *μ* = 0.3 on the corresponding optimal values of *γ*_0_, *γ*_1_, *γ*_2_ given in Table 3. From Figure 5 we see that, for *l* = 3, the value of ${\tilde{y}}_{3}(x,t)$ increases as we increase the value of *t*, whereas for *l* = 6, *l* = 8, and *l* = 10, the value of ${\tilde{y}}_{3}(x,t)$ decreases as we increase the value of *t*. Figures 6(a)–6(d) show the solution profile ${\tilde{y}}_{3}(x,t)$ versus *x* for *l* = 3, *l* = 6, *l* = 8, and *l* = 10, respectively, at *α* = 0.5, *μ* = 0.3 on the corresponding optimal values of *γ*_0_, *γ*_1_, *γ*_2_ given in Table 3. From Figure 6 we also find the same behaviour of ${\tilde{y}}_{3}(x,t)$ as in Figure 5.

**Figure 7:**
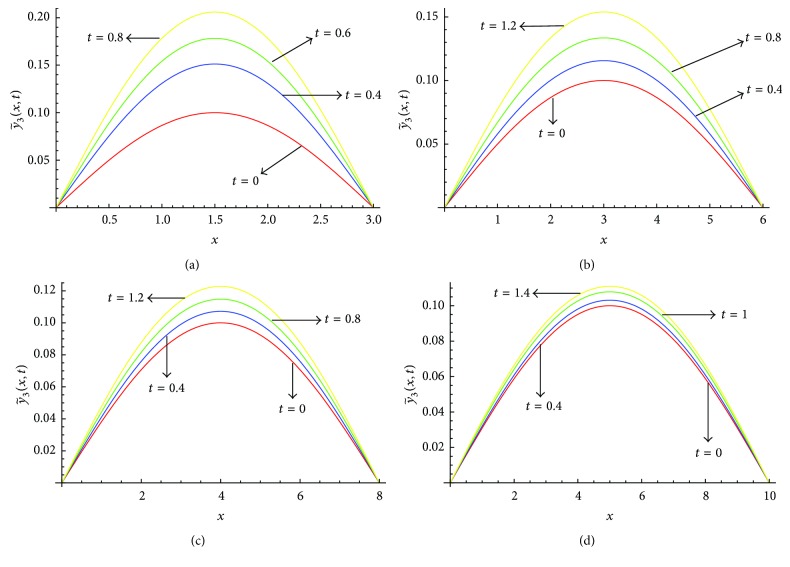
(a) Profiles of ${\tilde{y}}_{3}(x,t)$ versus *x* at *α* = 1, *μ* = 0.9 for *l* = 3 on the corresponding values of *γ*_0_, *γ*_1_, *γ*_2_ as given in Table 3. (b) Profiles of ${\tilde{y}}_{3}(x,t)$ versus *x* at *α* = 1, *μ* = 0.9 for *l* = 6 on the corresponding values of *γ*_0_, *γ*_1_, *γ*_2_ as given in Table 3. (c) Profiles of ${\tilde{y}}_{3}(x,t)$ versus *x* at *α* = 1, *μ* = 0.9 for *l* = 8 on the corresponding values of *γ*_0_, *γ*_1_, *γ*_2_ as given in Table 3. (d) Profiles of ${\tilde{y}}_{3}(x,t)$ versus *x* at *α* = 1, *μ* = 0.9 for *l* = 10 on the corresponding values of *γ*_0_, *γ*_1_, *γ*_2_ as given in Table 3.

**Figure 8:**
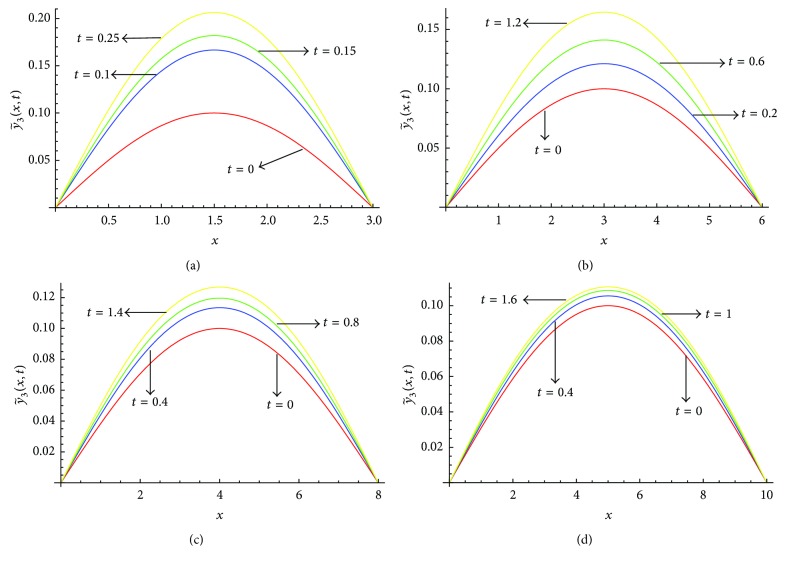
(a) Profiles of ${\tilde{y}}_{3}(x,t)$ versus *x* at *α* = 0.5, *μ* = 0.9 for *l* = 3 on the corresponding values of *γ*_0_, *γ*_1_, *γ*_2_ as given in Table 3. (b) Profiles of ${\tilde{y}}_{3}(x,t)$ versus *x* at *α* = 0.5, *μ* = 0.9 for *l* = 6 on the corresponding values of *γ*_0_, *γ*_1_, *γ*_2_ as given in Table 3. (c) Profiles of ${\tilde{y}}_{3}(x,t)$ versus *x* at *α* = 0.5, *μ* = 0.9 for *l* = 8 on the corresponding values of *γ*_0_, *γ*_1_, *γ*_2_ as given in Table 3. (d) Profiles of ${\tilde{y}}_{3}(x,t)$ versus *x* at *α* = 0.5, *μ* = 0.9 for *l* = 10 on the corresponding values of *γ*_0_, *γ*_1_, *γ*_2_ as given in Table 3.

Figures 7(a)–7(d) show the solution profile ${\tilde{y}}_{3}(x,t)$ versus *x* for *l* = 3, *l* = 6, *l* = 8, and *l* = 10, respectively, at *α* = 1, *μ* = 0.9 on the corresponding optimal values of *γ*_0_, *γ*_1_, *γ*_2_ given in Table 3. From Figure 7 we see that the value of ${\tilde{y}}_{3}(x,t)$ increases as we increase the value of *t*, for all considered values of *l*. Figures 8(a)–8(d) show the solution profile ${\tilde{y}}_{3}(x,t)$ versus *x* for *l* = 3, *l* = 6, *l* = 8, and *l* = 10, respectively, at *α* = 0.5, *μ* = 0.9 on the corresponding optimal values of *γ*_0_, *γ*_1_, *γ*_2_ given in Table 3. From Figure 8 we also find the same behaviour of ${\tilde{y}}_{3}(x,t)$ as in Figure 7.

## 4. Conclusion

We have proposed, for the first time, a new concept which is the generalization of our previous new iterative method [5] by introducing arbitrary number of auxiliary parameters and functions in the correction functional of the previously proposed new iterative method [5]. The method is called the optimal variational asymptotic method. A semianalytic algorithm based on OVAM is developed to solve fractional nonlinear partial differential equations. To test the algorithm, we apply it to solve two important classes of nonlinear partial differential equations: (1) the fractional advection-diffusion equation with nonlinear source term and (2) the fractional Swift-Hohenberg equation. Only five and three iterations are required to achieve fairly accurate solutions of the first and second problems, respectively. From Figures 1, 2, and 3, we see that OVAM is better than the methods in [5, 8]. Also, we get parabolic solution profiles for small and large values of *l* in 0 ≤ *t* ≤ 1.6 similar to the ones obtained in [35] whereas Vishal et al. [38] obtained hat like and nosey profiles for smaller values of *l* and the corresponding values of *μ*.

## Figures and Tables

**Table 1:** Optimal value of *γ*_0_, *γ*_1_,…, *γ*_4_ and the exact square residual error *J*_5_ for different values of *α*.

Auxiliary parameters	*γ*_0_	*γ*_1_	*γ*_2_	*γ*_3_	*γ*_4_	*J*_5_
*α* = 1	−1.10768	0.00350116	−0.000267365	0.0000205203	−1.25821 × 10^−6^	3.54123 × 10^−9^
*α* = 0.9	−1.14325	0.00505624	−0.000397341	0.0000255168	−1.96442 × 10^−7^	2.43911 × 10^−8^
*α* = 0.75	−1.22051	0.00813733	−0.000516221	−0.000020236	0.0000138436	4.16599 × 10^−7^
*α* = 0.5	−1.47194	0.0113722	0.00230399	−0.00101126	0.000107501	0.0000410035

**Table 2 tab2:** Comparison between our solution and that of Momani [8] for different values of *α*.

*x*	*α* = 0.5	*α* = 0.5 [8]	*α* = 0.75	*α* = 0.75 [8]	*α* = 0.9	*α* = 0.9 [8]	*α* = 1	*α* = 1 [8]
0.0	5.0083	5.00328	3.48585	3.48585	2.97494	2.97494	2.71828	2.71828
0.1	5.53503	5.52948	3.85246	3.85246	3.28782	3.28782	3.00417	3.00417
0.2	6.11715	6.11102	4.25763	4.25762	3.6336	3.6336	3.32012	3.32012
0.3	6.7605	6.75372	4.70541	4.70540	4.01575	4.01575	3.6693	3.6693
0.4	7.47151	7.46401	5.20028	5.20027	4.43809	4.43809	4.0552	4.0552
0.5	8.25729	8.24901	5.7472	5.74710	4.90484	4.90484	4.48169	4.48169
0.6	9.12572	9.11656	6.35164	6.35163	5.42069	5.42069	4.95303	4.95303
0.7	10.0855	10.07540	7.01964	7.01964	5.99079	5.99079	5.47395	5.47395
0.8	11.1462	11.13500	7.75791	7.75790	6.62085	6.62085	6.04965	6.04965
0.9	12.3184	12.30812	8.57381	8.57380	7.31717	7.31717	6.68589	6.68589
1.0	13.614	13.600031	9.47553	9.47552	8.08672	8.08672	7.38906	7.38906

**Table 3:** The optimal values of *γ*_0_, *γ*_1_, *γ*_2_ and the exact square residual error *J*_3_ for different values of *α*  , *l*, and *μ*.

Auxiliary parameters	*γ*_0_	*γ*_1_	*γ*_2_	*J*_3_
*α* = 1, *l* = 3, *μ* = 0.3	−0.130825	−0.143308	−0.537752	0.0000130186
*α* = 1, *l* = 6, *μ* = 0.3	−0.792741	−0.0301069	0.00643432	1.23122 × 10^−8^
*α* = 1, *l* = 8, *μ* = 0.3	−0.910664	−0.0000303478	0.000114225	4.64524 × 10^−10^
*α* = 1, *l* = 10, *μ* = 0.3	−0.897888	0.000353402	0.000163799	1.33347 × 10^−9^
*α* = 0.5, *l* = 3, *μ* = 0.3	−0.0816776	−0.180248	−0.667218	0.0000136652
*α* = 0.5, *l* = 6, *μ* = 0.3	−0.604212	−0.106513	0.0416011	1.07018 × 10^−7^
*α* = 0.5, *l* = 8, *μ* = 0.3	−0.819631	−0.00175609	0.000158241	2.87325 × 10^−9^
*α* = 0.5, *l* = 10, *μ* = 0.3	−0.795225	−0.00178556	0.000166566	4.72045 × 10^−9^
*α* = 1, *l* = 3, *μ* = 0.9	−0.22379	−0.366713	−0.294593	0.00169117
*α* = 1, *l* = 6, *μ* = 0.9	−0.87569	−0.0546794	0.0148106	2.05972 × 10^−8^
*α* = 1, *l* = 8, *μ* = 0.9	−1.08831	−0.00283652	−0.000322536	1.62026 × 10^−10^
*α* = 1, *l* = 10, *μ* = 0.9	−1.10053	−0.0114101	−0.00143646	1.70642 × 10^−11^
*α* = 0.5, *l* = 3, *μ* = 0.9	−0.121227	−0.338514	−1.16789	0.00317015
*α* = 0.5, *l* = 6, *μ* = 0.9	−0.623909	−0.50858	0.361561	2.46223 × 10^−7^
*α* = 0.5, *l* = 8, *μ* = 0.9	−1.18799	−0.0149661	−0.0022465	9.16058 × 10^−9^
*α* = 0.5, *l* = 10, *μ* = 0.9	−1.32112	−0.113086	−0.0418857	1.24439 × 10^−9^
*α* = 1, *l* = 10, *μ* = 0.6	−0.947507	−0.000676392	0.0000147138	1.42072 × 10^−11^
*α* = 0.75, *l* = 10, *μ* = 0.6	−0.927378	−0.00133351	0.0000116762	7.37925 × 10^−11^
*α* = 0.5, *l* = 10, *μ* = 0.6	−0.904097	−0.0028307	−0.0000115243	2.73053 × 10^−10^

## Appendix

We give some basic definitions and properties of fractional calculus [39] which have been used in the development of the proposed algorithm.

Definition 2 . A real function *f*(*x*), *x* > 0, is said to be in a space *C*_*μ*_, *μ* ∈ *R*, if there exists a real number *p*(<*μ*) such that *f*(*x*) = *x* ^*p*^ *f*_1_(*x*) where *f*_1_(*x*) ∈ *C*[0, *∞*), and it is said to be in the space *C*_*m*_^*μ*^ if *f* ^(*m*)^ ∈ *C*_*μ*_, *m* ∈ *N*.

Definition 3 . The Riemann-Liouville fractional integral operator of order *α* ≥ 0, of a function *f* ∈ *C*_*μ*_, *μ* ≥ −1, is defined as

(A.1) $$\begin{array}{c} J_{a}^{\alpha }f\left(x\right)=\frac{1}{\Gamma \left(\alpha \right)}\overset{x}{\underset{a}{\int }}{\left(x-t\right)}^{\alpha -1}f\left(t\right)dt,\;\alpha >0,\;\;x>0. \end{array}$$

For

(A.2) $$\begin{array}{c} \alpha ,\beta >0,\;a\geq 0,\;\gamma \geq -1,\\ J_{a}^{\alpha }{\left(x-a\right)}^{\gamma }=\frac{\Gamma \left(1+\gamma \right)}{\Gamma \left(1+\gamma +\alpha \right)}{\left(x-a\right)}^{\gamma +\alpha }. \end{array}$$

Definition 4 . The fractional derivative of order *α* of a function *f*(*x*) in Caputo sense is defined as

(A.3) Daαf(x)=Jam−αDaαf(x)=1Γ(m−α)∫ax(x−t)m−α−1fm(t)dt

for *m* − 1 < *α* ≤ *m*, *m* ∈ *N*, *x* > *a*, *f* ∈ *C*_−1_^*m*^. The following properties of the operator *D*_*a*_^*α*^ are well known:

(A.4) $$\begin{array}{c} D_{a}^{\alpha }J_{a}^{\alpha }f\left(x\right)=f\left(x\right),\\ J_{a}^{\alpha }D_{a}^{\alpha }f\left(x\right)=f\left(x\right)-\overset{m-1}{\underset{k=0}{\sum }}f^{\left(k\right)}\left(a\right)\frac{{\left(x-a\right)}^{k}}{k!},\;x>0. \end{array}$$
